# Histoplasmosis in a State Where It Is Not Known to Be Endemic — Montana, 2012–2013

**Published:** 2013-10-25

**Authors:** Donald Skillman, Laurel Riek, Brian Davis, Julie R. Harris, Randall J. Nett

**Affiliations:** St. Peter’s Medical Group, Helena; Lewis and Clark City-County Health Dept, Helena; Billings Clinic, Billings, Montana; Div of Foodborne, Waterborne, and Environmental Diseases, National Center for Zoonotic, Vector-Borne, and Enteric Diseases; Career Epidemiology Field Officer Program, Div of State and Local Readiness, CDC

Histoplasmosis is caused by infection with the dimorphic fungus, *Histoplasma capsulatum*, following inhalation of contaminated soil (1). Among symptomatic patients, the most common clinical presentation is acute pneumonia (1–3). Persons with compromised immune systems are at risk for disseminated histoplasmosis, a severe illness requiring antifungal therapy that is often characterized by fever, malaise, anorexia, and weight loss (2,4). *H. capsulatum* is endemic in the Ohio River and Mississippi River valleys, where it is found in soil enriched with bird droppings and bat guano (1,5–7). During November 2012–February 2013, histoplasmosis was diagnosed in four Montana residents by four different physicians. No epidemiologic links among the cases were identified. Each patient’s medical records were reviewed, and their exposure and travel histories were obtained. Three patients reported no recent travel outside of Montana and likely were exposed in Montana, which is west of areas where *H. capsulatum* is recognized as endemic (5,6). One patient reported recent travel to California, where she was exposed to potting soil containing bat guano. Low clinical suspicion, probably related to lack of history of exposure to areas where *H. capsulatum* is known to be endemic, likely delayed diagnosis and appropriate therapy for three patients. Health-care providers should be aware of the possibility of histoplasmosis in Montana and consider the diagnosis in patients with clinically compatible illnesses.

## Patient 1

A woman aged 59 years with a history of breast cancer who was treated with chemotherapy and radiation in 2002 was evaluated in February 2011 for left parotid gland swelling (Table). A computed tomography (CT) scan with intravenous (IV) contrast revealed a 2-cm lesion in the tail of the parotid gland. On April 6, the patient underwent a fine needle aspiration of her left parotid gland; cytology revealed atypical lymphoepithelial cells. In October 2012, the patient reported continued swelling behind her left ear, which eventually developed into a weeping lesion. She reported no fever, chills, night sweats, weight loss, cough, or difficulty breathing. On November 11, she underwent a left parotidectomy. Histopathology revealed lymphoepithelial cysts and necrotizing granulomatous inflammation with rare yeast forms consistent with *H. capsulatum*. A follow-up urine histoplasma antigen enzyme immunoassay (EIA) on December 7, 2012, was negative. The patient was referred for treatment of histoplasmosis and started on itraconazole therapy.

The patient was a retired administrator and had never lived outside southwest Montana. She reported engaging in walking, fishing, and gardening, and had multiple bird feeders. She did not report any exposures to caves or bats. In May 2012, the patient visited Sacramento, California, where she participated in the potting of plants using soil labeled as containing bat guano. No other persons exposed to the same potting soil during her trip were known to have become ill. Other than the trip to Sacramento, the patient had not traveled outside of Montana for several years.

## Patient 2

An adolescent boy aged 17 years sought care in November 2011 with complaints of fatigue. Following a positive heterophile antibody test for Epstein-Barr virus, he was diagnosed with mononucleosis. Over the next 9 months, the patient continued to experience fatigue, headaches, and night sweats, and had a 5-pound (2.3 kg) weight loss. In September 2012, he sought follow-up care and was noted to have clinically significant anterior cervical lymphadenopathy. On September 19, a CT scan of the pelvis with IV contrast revealed extensive mesenteric and bilateral inguinal lymphadenopathy. At that time, a chest radiograph, complete blood count, electrolytes, and liver function tests were within normal limits. His Epstein-Barr virus immunoglobulin (Ig)M was 11.7 U/mL (normal = 0–43.9 U/mL) and IgG was 539.0 U/mL (normal = 0–21.9 U/mL). On December 27, he underwent a lymph node biopsy of an anterior cervical lymph node and a submandibular node that revealed necrotizing granulomatous inflammation with yeast forms consistent with *H. capsulatum*. A follow-up urine EIA on January 15, 2013, was negative. The patient was started on itraconazole therapy.

The patient had lived in southwest Montana since birth. He reported frequent outdoor recreational activities, including exploring caves, trundling (rolling large rocks or boulders down hillsides), wakeboarding, camping, hiking, and snowboarding. He also worked as a landscaper during the summer of 2012. He did not report any known exposures to bats or bird droppings. His last travel outside of Montana was to Omaha, Nebraska, in 2007.

## Patient 3

A man aged 79 years with a history of uncontrolled diabetes mellitus type II, tobacco use, and colon cancer treated with a partial colectomy in 2003 experienced fatigue and fever on July 3, 2012, and was evaluated in an emergency department on July 8; a chest radiograph was normal. Because of persistent fevers noted when the patient was reevaluated on July 18, the patient underwent a CT scan of the chest, abdomen, and pelvis without IV contrast; the scan revealed a left lower lobe pneumonia and mild pulmonary fibrosis of the right lung base. Laboratory analysis of a blood specimen revealed a mild leukocytosis and an erythrocyte sedimentation rate of 81 mm/hr (normal: 0–20 mm/hr). The patient was started on moxifloxacin 400 mg daily for 10 days. On reevaluation on July 25, the patient was noted to be afebrile and clinically improved. On October 10, the patient underwent a follow-up CT scan of the chest without IV contrast, which revealed a 3 cm by 1.3 cm oval opacity in the posterior left lower lung and a 4 mm high-density nodule in the right lower lung consistent with a calcified granuloma. At this time, the patient’s leukocytosis had resolved, and erythrocyte sedimentation rate was 42 mm/hr. In November 2012, the patient underwent a CT-guided biopsy of the left lower lung lesion that demonstrated a granulomatous pneumonitis.

In December 2012, a positron emission tomographic scan of the chest demonstrated mediastinal and subcarinal lymphadenopathy and a left lung nodule that had increased glucose uptake. The patient then underwent mediastinoscopy with tissue biopsy. In February 2013, the biopsy specimen was culture-positive for *H. capsulatum*. The patient was referred for treatment of histoplasmosis.

The patient was a rancher and lived in eastern Montana. He reported travel >30 years earlier to Canada and Europe but reported no travel since that time outside of eastern and south central Montana. During the 2 weeks before illness onset, he reported mowing grass in a pasture and attending a local rodeo. He did not report any exposures to bird droppings, bat guano, caves, or potting soils.

## Patient 4

A woman aged 76 years with a history of uncontrolled diabetes mellitus type II experienced nasal congestion, nonproductive cough, headaches, chest pressure, shortness of breath, and wheezing on January 17, 2013. Four days later, the patient was evaluated in an emergency department. She did not report any fever or myalgias. On examination, she was noted to be afebrile, had bilateral expiratory wheezing, and was hypoxic with a pulse oximetry oxygen saturation of 77% on room air. The patient tested positive for influenza A using a rapid diagnostic test and was hospitalized for additional care. She underwent CT scans of the chest, abdomen, and pelvis with IV contrast, which revealed bibasilar patchy infiltrates along with retroperitoneal and mesenteric lymphadenopathy and rectal wall thickening. The patient’s urine tested positive for histoplasma antigen by EIA. When discharged from the hospital, she was referred for treatment of histoplasmosis, and started on itraconazole therapy.

The patient lived in southwestern Montana and was a retired custodian. She enjoyed reading indoors, and engaged in no outdoor activities other than planting flowers in outdoor flowerpots each spring. She denied known exposures to bird droppings, bat guano, bat guano-containing potting soil, or caves. She reported no travel outside of Montana in the several years before her diagnosis.

### Editorial Note

Three of four patients with diagnosed histoplasmosis reported no recent travel and likely acquired their infections in Montana. Although patient 1 likely acquired her infection in Montana before traveling out of state, the possibility also exists that she acquired infection in California following exposure to bat guano–containing potting soil. Each of the four patients had immunocompromising conditions present before symptom onset, increasing their risk for *H. capsulatum* disease (2). Patient 2 might have acquired infection during a cave exploration–related bat guano exposure. The lack of recent travel history to recognized areas with histoplasmosis endemicity likely contributed to diagnostic delays for three patients; of these, two patients also had unusual clinical presentations, likely further contributing to diagnostic delays.

*H. capsulatum* culture from body fluids and tissues provides the strongest evidence of histoplasmosis, but is insensitive (8). Patient 3 was diagnosed after *H. capsulatum* isolation from a pulmonary nodule biopsy. The absence of recent travel outside of Montana for this patient suggests that the infection was acquired in Montana. Clinical evidence for patients 2 and 4 suggests their *Histoplasma* infections also were acquired in Montana.

Patients 1 and 2 were diagnosed by histopathology performed by different pathologists. Histopathology provides presumptive evidence of infection, but specificity depends on pathologist experience (8). Urine antigen detection also is used to diagnose disseminated histoplasmosis (2,8,9), but its sensitivity is greater among immunocompromised persons (93.1% compared with 73.3%) (9). The specificity of urine EIA for histoplasmosis has been shown to be 99% in both healthy and immunocompromised persons (8,9), although false-positives are possible among patients with other endemic fungal infections (2). Why patients 1 and 2 had negative urine EIA tests following histopathologic diagnoses is unclear. Although patient 2 likely was immunocompromised when he developed disease, his immune system might have recovered by the time of EIA testing.

What is already known on this topic?

Histoplasmosis is a potentially severe illness caused by infection with the dimorphic fungus *Histoplasma capsulatum*. This fungus is endemic in the Ohio River and Mississippi River valleys in the United States, where it is found in soil enriched with bird droppings and bat guano.

What is added by this report?

Four unrelated cases of histoplasmosis were identified during 2012–2013 among residents of Montana, a location further west than areas where *H. capsulatum* is known to be endemic.

What are the implications for public health practice?

Health-care providers should be aware of the possibility of *H. capsulatum* in Montana and the potential for histoplasmosis in patients with clinically compatible illnesses, even in the absence of a history of travel outside of the state.

The recognized zone of histoplasmosis endemicity extends from the Ohio River Valley west into North Dakota and South Dakota (6). States further west, including Montana, are not typically considered areas where histoplasmosis is endemic. However, two recently published studies provide limited data that histoplasmosis endemicity might extend into Montana and other western states (5,10). The four cases described in this report provide additional evidence that histoplasmosis should be considered in the differential diagnosis of clinically compatible illness in Montana residents.

The findings in this report are subject to at least two limitations. First, confirmation of the diagnosis by isolation of *H. capsulatum* in culture was not performed for three patients. Second, the source of each patient’s infection could not be determined with certainty; patient 1 likely acquired her infection in Montana, but also could have acquired her infection from exposure to bat guano–containing potting soil in California. Similarly, one or more patients might have had reactivation of latent disease after *H. capsulatum* infection acquired from an area when histoplasmosis was endemic many years before symptom onset. Environmental studies could help determine if *H. capsulatum* is endemic in Montana (5,6).

Three patients experienced diagnostic delays, likely in part because none reported recent travel to areas where *H. capsulatum* is endemic. Health-care providers should be aware of the possibility of *H. capsulatum* in Montana and the potential for histoplasmosis in patients with clinically compatible illnesses, even in the absence of a history of travel outside of Montana.

## Figures and Tables

**TABLE:** Characteristics of four patients with diagnosed histoplasmosis — Montana, 2012–2013

Patient	Age (yrs)	Sex	Area of residence	Month of symptom onset	Clinical presentation	Immuno- compromising condition	Laboratory testing*	Site of infection	Recent travel outside Montana†	Possible high-risk exposures
1	59	F	Southwest Montana§	February 2011¶	Left parotid gland swelling	Yes	Histopathology = positive; EIA = negative	Disseminated	Yes: California (May 2012)	Bat guano–containing potting soil (May 2012)
2	17	M	Southwest Montana§	November 2011**	Fatigue, night sweats, weight loss, cervical lymphadenopathy	Yes	Histopathology = positive; EIA = negative	Disseminated	No	Exploring caves, water sports on lakes and rivers
3	79	M	East Montana	July 2012	Fever, fatigue, pneumonia	Yes	Culture = positive	Pulmonary	No	None known
4	76	F	Southwest Montana§	January 2013	Headache, cough, wheezing, hypoxia	Yes	EIA = positive	Disseminated	No	None known

**Abbreviation:** EIA = enzyme immunoassay.

* Laboratory tests that were not performed (i.e., histopathology, EIA, serology, and culture) are not included in the table.

† Defined as travel outside of Montana since 2008.

§ The three patients in southwest Montana lived within a 15-mile (24-km) radius.

¶ Patient 1 first noticed left parotid gland swelling in February 2011 but continued to have clinical illness through November 2012.

** Patient 2 received an initial diagnosis of acute mononucleosis in November 2011 but continued to have clinical illness through December 2012.
